# Contribution of glutathione peroxidase 1 (Pro200Leu) single nucleotide polymorphism and serum homocysteine levels in the risk of acute myocardial infarction in Egyptians

**DOI:** 10.1186/s43141-022-00307-6

**Published:** 2022-02-09

**Authors:** Lamia K. Ismail, Mohamed F. Abdel Rahman, Ingy M. Hashad, Sahar M. Abdel-Maksoud

**Affiliations:** 1 Clinical Biochemistry Department, Faculty of Pharmacy and Biotechnology, the German University, Cairo, Egypt; 2 Department of Biology and Biochemistry, School of Life and Medical Sciences, University of Hertfordshire Hosted by Global Academic Foundation, Cairo, Egypt

**Keywords:** Acute myocardial infarction, Oxidative stress, Glutathione peroxidase 1, Homocysteine, Single nucleotide polymorphism

## Abstract

**Background:**

Oxidative stress is among the most common risk factors in the pathogenesis of acute myocardial infarction (AMI). Glutathione peroxidase 1 enzyme coded by the *GPX1* gene plays an essential role in reducing oxidative stress. Previous studies correlated the *GPX1* (Pro200Leu) single nucleotide polymorphism (SNP) with AMI incidence. Elevated homocysteine (Hcy) levels induce oxidative stress and are considered an independent risk factor for AMI. Evidence showed a complex relationship between Hcy and GPx-1 activity. This study examined the association of the common (Pro200Leu) SNP in *GPX1* with AMI incidence in an Egyptian population. This study is the first to check this association in an Egyptian population. Moreover, the association between serum Hcy and the incidence of AMI was checked, and the novelty was to statistically correlate *GPX1* Pro200Leu genotypes with serum Hcy levels in patients and control subjects. Hundred control subjects and hundred and twenty AMI patients were genotyped using PCR-RFLP analysis. An ELISA was used to measure serum Hcy levels.

**Results:**

The *GPX1* (Pro200Leu) genotype distribution and allele frequency were not significantly different between patients and control subjects (*P* = 0.60 and *P* = 0.62, respectively). Serum levels of Hcy were significantly elevated in patients compared to control subjects (*P* ≤ 0.0001). However, no significant difference was observed in serum Hcy levels among different *GPX1* genotypes in neither patients nor control subjects.

**Conclusions:**

The minor T allele of *GPX1* Pro200Leu is not associated with AMI risk in this Egyptian population. However, high homocysteine serum levels might contribute independently to the risk of AMI. Finally, Hcy levels were not significantly different in homozygous minor TT compared to homozygous wild CC.

## Background

Cardiovascular diseases (CVDs) are the pre-eminent cause of death globally. They are significant health problems, not only due to high incidence but also due to the socioeconomic burden associated with them. Acute myocardial infarction (AMI) is one of the most common CVDs that cause death worldwide [1]. The initial trigger of AMI is mainly irreversible myocardial necrosis that is secondary to prolonged ischemia. The disparity between the myocardial blood supply and demand leads to ischemia [2]. Previous studies showed the involvement of oxidative stress-mediated by reactive oxygen species (ROS) in the pathogenesis of AMI. The exact mechanism by which ROS contributes to AMI is still uncertain; however, the most common hypothesized mechanism is that ROS, mainly hydrogen peroxide (H_2_O_2_), leads to nitric oxide (NO) insufficiency by converting it to inactive peroxynitrite. NO is the most potent vasodilator. NO also alters the adherence of the platelets and leukocytes to the endothelial membrane and improves the barrier function of the endothelium [3].

The glutathione peroxidase (GPx) family are the fundamental antioxidant enzymes in humans [4]. The GPx family consists of eight isoforms named from GPx-1 to GPx-8; each isoform has distinct subcellular localization. The principal role of these enzymes is to convert hydrogen and lipid peroxides to their reduced form, water (H_2_O) and alcohol (LOH), respectively. Glutathione peroxidase 1 (GPx-1) is the most abundant intracellular isoform [4–6]. The cardioprotective role of GPx-1 is manifested by preventing oxidative stress-induced atherosclerosis [7, 8]. This is attained by reducing the availability of ROS, so low-density lipoprotein (LDL) becomes less prone to be oxidized to oxidized LDL (Ox-LDL), which is the fundamental component of the atherosclerotic plaque and the necrotic core [8, 9]. Furthermore, it regulates NO bioavailability by decreasing the levels of hydrogen and lipid peroxides, hence decreasing the susceptibility of NO inactivation to peroxynitrite [10]. The *GPX1* gene maps chromosome 3p21. The Pro200Leu single nucleotide polymorphism (SNP) (rs1050450) is a missense mutation that occurs on exon two where the wild allele C is substituted by the T allele (NM_000581.4:c.599C>T) resulting in the amino acid change of Pro to Leu [6, 11] that impairs the enzyme activity [12, 13]. This SNP was previously associated with CVD risk in various ethnicities [14–17].

Homocysteine (Hcy) is a non-essential amino acid. It shows structural homology to cysteine amino acid yet with an additional methylene bridge (-CH2-) [18]. The physiological role of Hcy is debatable and not well known, unlike its toxicity which is mainly due to the covalent interaction between Hcy and the proteins which alter their functions [19]. Elevated Hcy levels are known as hyperhomocysteinemia (HHcy) [20]. Several studies showed a correlation between HHcy and oxidative stress [19, 21]. HHcy-induced oxidative stress can be classified into direct and indirect mechanisms. The direct mechanism involves Hcy being auto-oxidized to homocysteine, releasing H_2_O_2_ as a by-product [22], while the indirect mechanisms involve HHcy inducing ROS generation in several ways. HHcy leads to uncoupling of eNOS and increases xanthine oxidase and NADPH oxidase activity [23–25]; all lead to an increase in superoxide anion generation. The indirect mechanism also involves HHcy downregulating several antioxidant enzymes, mainly GPx-1, as well as depleting its substrate glutathione [10, 26, 27], hence decreasing the antioxidant effect and increasing the oxidative stress. Several experimental and clinical evidence has demonstrated a unique and complex relationship between Hcy and GPx-1 activity. Extreme HHcy inhibited GPx-1 activity in both in vivo and in vitro [28] by a mechanism involving downregulation of translation, which may contribute to the proatherogenic and prothrombic effects of Hcy [26]. Also, individuals with high GPx-1 activity are less susceptible to Hcy damaging effects [29].

Given the above findings, this study examined the association between *GPX1* Pro200Leu common SNP and the risk of AMI in an Egyptian population, besides the correlation between serum Hcy levels and the risk of AMI, in addition to statistically correlating *GPX1* Pro200Leu different genotypes with the levels of Hcy in control subjects and AMI patients.

## Methods

### Study subjects

Hundred and twenty AMI patients were recruited for this study. The blood samples were collected from patients admitted to the intensive care unit of the National Heart Institute, Imbaba, Giza; El Demerdash Hospital, Cairo, Egypt. All recruited patients were unrelated. Patients were only included if they have been diagnosed with AMI for the first time, confirmed by electrocardiogram and elevated cardiac markers. The samples were drawn within the first 6 h from the myocardial infarction episode. The patients were divided into fifty-five females with an age range of 46–60 years and sixty-five males with an age range of 44–60 years.

For the control subjects, hundred healthy individuals were included in this study. All individuals were unrelated, and the samples were collected from the blood bank of 57357 Hospital, Cairo, Egypt. The individuals included forty females with an age range of 41–57 years and sixty males with an age range of 42–58 years. Subjects were only included in this study if they had no history of AMI or any other cardiovascular diseases besides having a controlled blood pressure of 120/70 mmHg.

The exclusion criteria included any other acute or chronic diseases such as diabetes mellitus, renal or hepatic diseases, cancer, or any other CVDs. This criterion was applied to all study participants. The participants filled out medical reports, which were used to obtain information regarding their family history and lifestyle.

All the procedures comply with the ethical standards of German University in Cairo ethics committee and the 1964 Helsinki Declaration. Written consent was obtained from all study participants.

### Sample collection

Four milliliters of blood samples was collected. The samples were allowed to clot at room temperature for 30 min [30] followed by centrifugation at 2500 rpm for 10 min at 4 °C to obtain the serum. The serum was then stored at −20° C for Hcy determination.

Whole blood was used for DNA extraction using DNA Blood GeneJET Mini Kits (Thermo Scientific). The extraction was carried out based on the manufacturer’s instructions. Briefly, proteinase K and lysis buffer were added to the blood sample to break down nuclear and cellular membranes, releasing the DNA. The released DNA was then allowed to bind specifically to the silica-gel membrane of the GeneJET mini spin column while contaminants were washed through. The DNA was eluted via elution buffer provided with the kit. Then, the purity of the eluted DNA was assessed using Nanodrop and was quantified using Qubit. The extracted, pure DNA was used for genotyping the *GPX1* Pro200Leu SNP using polymerase chain reaction-restriction fragment length polymorphism (PCR-RFLP) [31].

### The GPX1 Pro200Leu SNP genotyping by PCR-RFLP

**Fig. 1:**
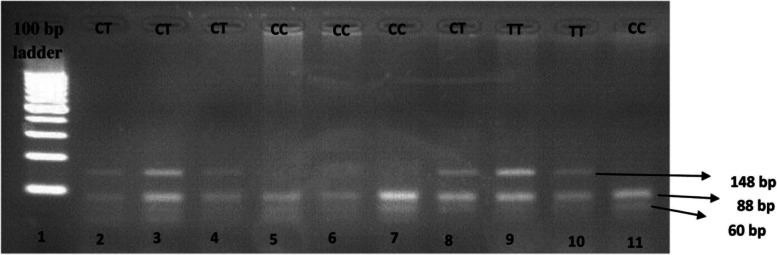
The gel shows all the possible genotypes. The samples (50 ng) were loaded and ran against a 100-bp DNA ladder marker. In the case of homozygous wild CC as in lanes 5, 6, 7, and 11, 3 bands were obtained (88, 82, and 60bps). In the case of heterozygous CT as in lanes 2, 3, 4, and 8, 4 bands were obtained (148, 88, 82, and 60bps), while for homozygous minor TT as in lanes 9 and 10, only 2 bands were obtained (148 and 82bps)

The forward primer used for the amplification of the 230-bp fragment was 5′-TTATGACCGACCCCAAGCTCA-3′ while the reverse primer was 5′-ACAGCAGCACTGCAACTGCC-3′ [32]. A 1 μl of *Hae* III restriction enzyme was used to digest the obtained PCR product. The PCR digests were then loaded on 3% agarose gel and visualized under UV after staining with ethidium bromide. In the case of the minor allele T, 148 and 82bp band sizes are obtained while for the wild allele C, 88, 82, and 60bp bands are obtained as shown in Fig. 1.

### Measuring serum Hcy concentrations

Serum Hcy levels were quantified using the human Hcy enzyme-linked immunosorbent assay (ELISA) kit provided by Axis-Shield, Dundee, UK.

### Lipid profiling for study participants

The serum samples were also used to measure the levels of triglycerides (TG) and total cholesterol (TC) via an enzymatic colorimetric method using kits provided by Diamond diagnostics, Egypt.

### Statistical analysis

All the statistical analyses were performed using GraphPad Prism software (GraphPad Software, Inc). All continuous data were presented as median (IQR). The differences between the two study groups were assessed using either the nonparametric student *t*-test (Mann-Whitney) or the nonparametric one-way ANOVA (Kruskal-Wallis). The odds ratio (OR) with 95% confidence interval (CI) were used to check whether the minor allele T is associated with increased risk of AMI or not. A two-tailed *P-*value ≤ 0.05 was used as the significance threshold for all tests. However, *P* > 0.05 indicated no deviation from the Hardy-Weinberg equilibrium (HWE).

## Results

### Demographics of the study cohort

The demographics of the study cohort, serum TC, TG, and Hcy levels are shown in Table 1.

**Table 1:** Demographics of the study participants

Groups	Control subjects	AMI patients
**Number (male/female)**	100 (60/40)	120 (65/55)
**Age range**	41–58	44–60
**Serum total cholesterol (mg/dL)**	175 (23)	222 (59)
**Serum triglycerides (mg/dL)**	108 (44)	149 (67)
**Serum homocysteine (μmole/L)**	15 (5.5)	29 (9.5)

Categorical data are presented as number. Continuous data are presented as median (IQR)

### Genotyping of the Pro200Leu SNP

The pattern of the genotype distribution was not significantly different between AMI patients and control subjects (Mann-Whitney test, *P* = 0.60). Similarly, there was no significant difference in the allele frequencies between the two groups (Mann-Whitney, *P* = 0.62). Carriers of the risk allele T allele (CT+TT) did not show a higher risk for incidence of AMI compared to wild CC genotype (*OR* = 0.8623; *P* = 0.5862), as illustrated in Table 2.

**Table 2:** Odds ratio (OR) with 95% confidence intervals (CI) for *GPX1* Pro200Leu in study groups

***GPX1*** Pro200Leu	AMI patients (***n*** = 120)	Control subjects (***n*** = 100)	***OR*** (95% ***CI***)	***P-***value
**CT+TT**	52	45	0.8623 (0.5058–1.470)	0.5862
**CC**	68	55		

### Levels of Hcy in serum

**Fig. 2:**
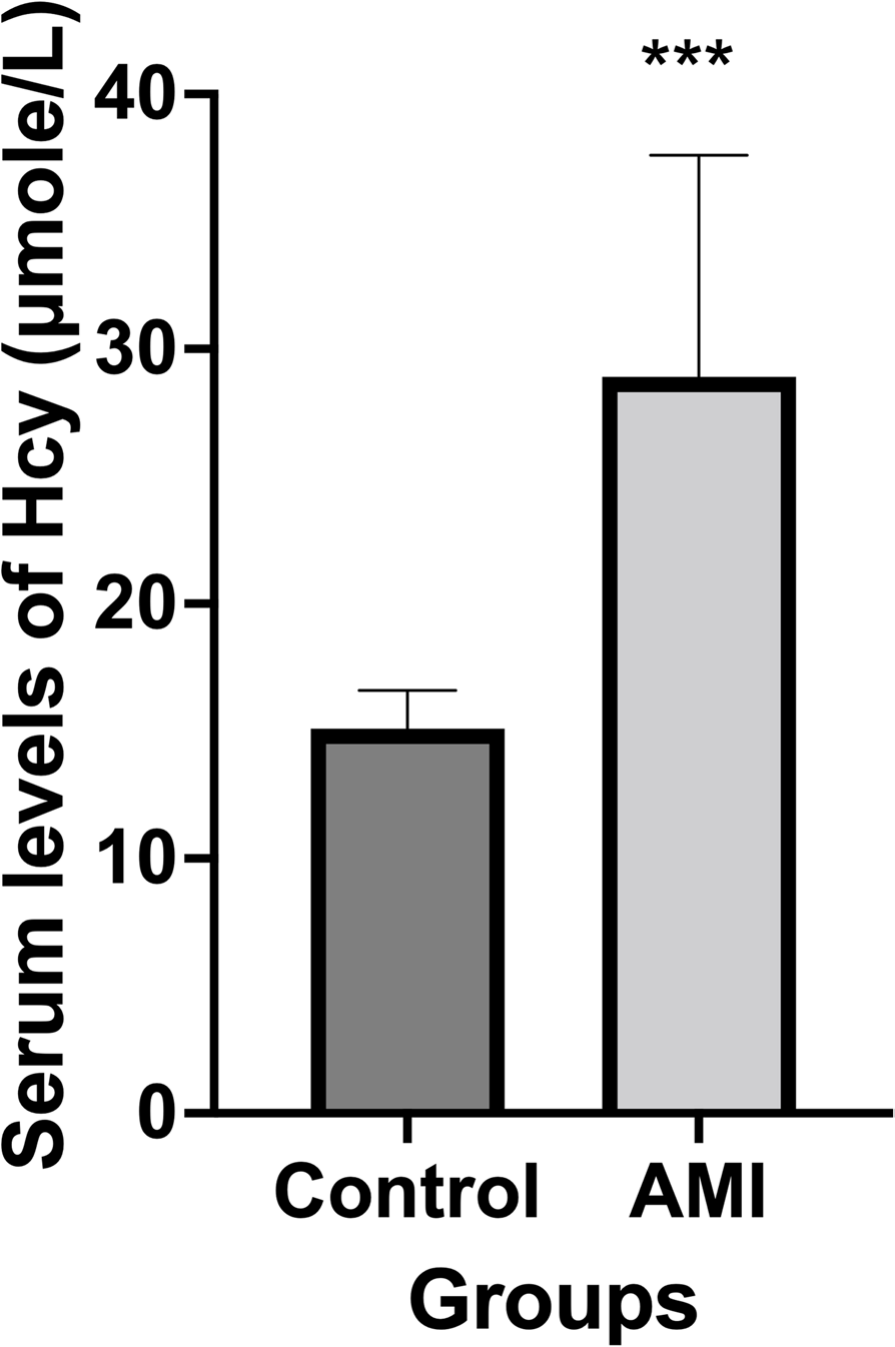
The median serum Hcy levels in both study groups. ***Significant difference from the control group at *P*≤ 0.0001

The AMI group had up to a 1.90-fold significant increase in median serum levels of Hcy compared to control subjects (*P* ≤ 0.0001) as shown in Fig. 2.

### Correlation of genotypes with serum Hcy levels in study groups

**Fig. 3:**
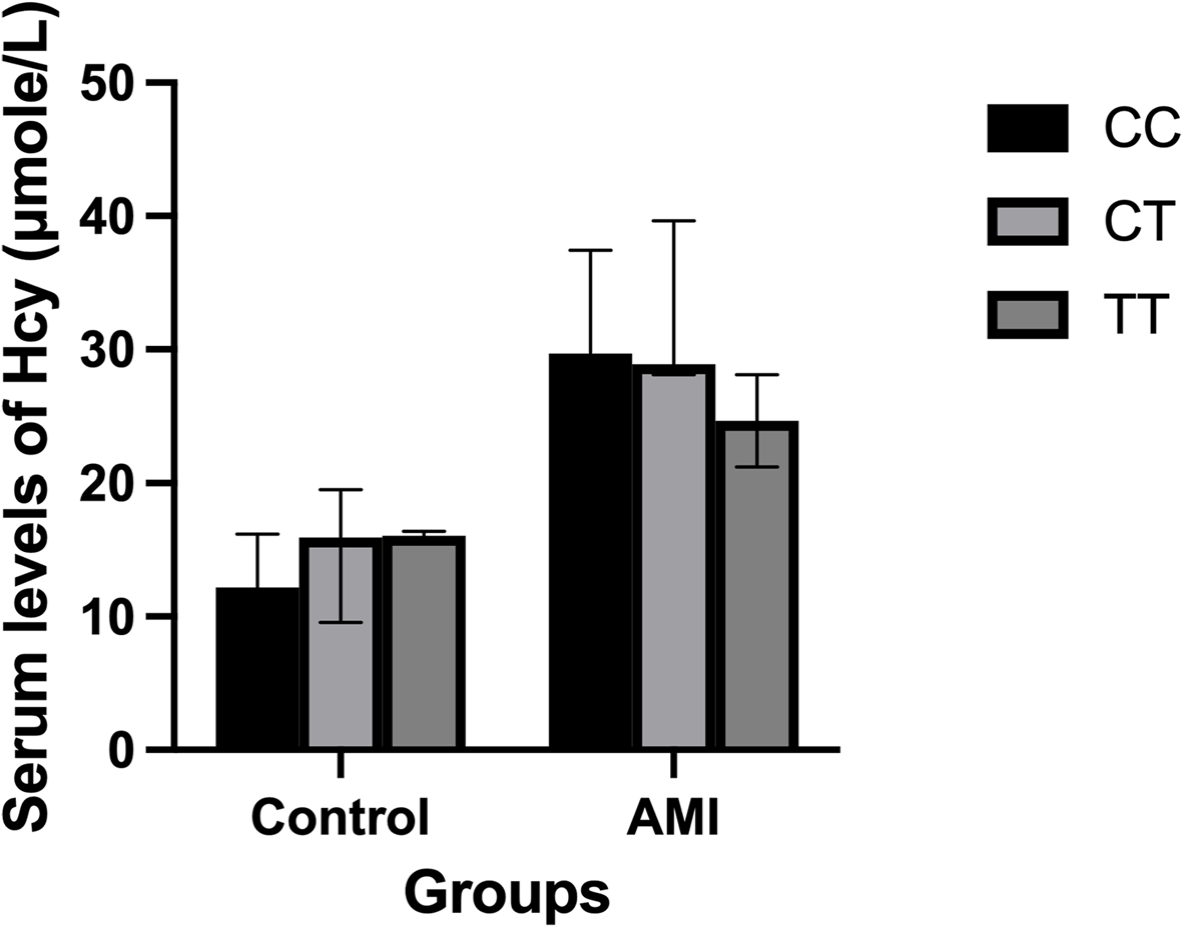
The median serum Hcy concentrations among all genotypes in both control and AMI

There was no significant correlation between different genotypes and median serum Hcy levels in neither AMI patients nor control subjects (*P* = 0.186 and *P* = 0.373, respectively) as shown in Fig. 3.

### TG and TC levels between study groups

The AMI group had up to 1.38-fold significant increase in the median serum TG concentration compared to controls (*P* < 0.0001). Similarly, a significant (*P* < 0.0001) increase up to 1.27-fold was observed in the median serum TC concentration in the AMI group compared to controls.

## Discussion

### Correlation between GPX1 Pro200Leu SNP and risk of AMI

Under normal physiological conditions, GPx-1 reduces oxidative stress tremendously [5]. Cheng et al. reported that patients with impaired GPx-1 activity are more prone to develop AMI [33]. As far as we know, this study is the first to clinically investigate the possible association of *GPX1* (Pro200Leu) SNP and the risk of AMI in an Egyptian population. The current study results showed no significant difference in neither the *GPX1* genotype distribution nor the allele frequency between AMI and control groups suggesting that the *GPX1* Pro200Leu SNP is not associated with the risk of AMI. Our results showed consistency with studies done on Chinese [14], Swedish [15], Indian [16], and Russian populations [17]. However, our results contrasted the one done on Japanese [11].

A suggested explanation for the above significant correlation between this SNP and AMI is that the Pro to Leu amino acid change could alter the activity of the enzyme [12] since the Leu variant was previously associated with a reduction in the enzyme activity [11, 34]. The GPx-1 enzyme is abundant in endothelial cells and macrophages; therefore, the decreased activity is expected to increase the sensitivity of the vessels to oxidative stress and will be more prone to oxidative stress-induced atherosclerosis [32]. The above conflicting findings may be attributed to the differences in ethnicities, trial numbers, different methods applied, sampling schemes, and the different environmental effects [26, 35].

### Serum Hcy levels

Hcy is a non-essential amino acid with an extra sulfur; the main pathway for Hcy synthesis is methionine demethylation [18]. Hcy levels are maintained via two pathways, either remethylation or trans-sulfuration [36]. Hcy levels above 16 μmole/L are known as HHcy [37] and were previously shown to be independently a risk factor for various diseases, including CVDs [38–40].

In this study, the median Hcy concentration was significantly higher in the AMI group compared to the control group (*P* ≤ 0.0001). This significant correlation can be due to several effects; Hcy triggers the endothelium to synthesize and release pro-coagulant factors [41]. It also enhances the auto-oxidation of LDL to Ox-LDL [42], promotes vascular thrombosis by reducing the activation of protein C, initiates the aggregation of the platelets, and stimulates smooth muscle cell proliferation [38, 43]. Our results were consistent with those observed in several other ethnicities [44–46].

### Correlation between SNP and Hcy in study subjects

Experimental evidence showed that GPx-1 regulates Hcy-induced cardiovascular risk and that Hcy attenuates the ability of the cell to detoxify hydrogen peroxide by inhibiting the intracellular activity of GPx-1 [29]. Hcy also downregulates translation [26]. Therefore, a possible correlation between the rs1050450 variant and serum Hcy levels was hypothesized.

In the current study, the *GPX1* Pro200Leu SNP and the Hcy serum levels were correlated in neither the AMI (*P* = 0.186) nor the controls (*P* = 0.373). Our study was the first to correlate the SNP and Hcy serum levels in humans. However, our results were consistent with Dayal et al.’s findings in mice in which Hcy levels were not associated with the *GPX1* genotypes [47].

## Conclusion

We can conclude from this study that *GPX1* Pro200Leu SNP was not significantly associated with AMI in an Egyptian population. However, Hcy’s role in the incidence of AMI was confirmed since AMI patients had significantly 1.90-fold higher median serum levels of Hcy compared to healthy control subjects, while the novel correlation between Hcy serum levels and the SNP showed no significant difference in neither healthy control subjects nor AMI patients.

## Data Availability

The datasets used and/or analyzed during the current study are available from the corresponding author on reasonable request.

## Abbreviations

AMI: Acute myocardial infarction
bp: Base pairs
CI: Confidence interval
CVDs: Cardiovascular diseases
DNA: Deoxyribonucleic acid
ELISA: Enzyme-linked immunosorbent assay
GPx: Glutathione peroxidase
GPx-1: Glutathione peroxidase 1
Hcy: Homocysteine
HHcy: Hyperhomocysteinemia
HWE: Hardy-Weinberg equilibrium
IQR: Inter-quartile range
LDL: Low-density lipoprotein
Leu: Leucine
NO: Nitric oxide
OR: Odds ratio
Ox-LDL: Oxidized low-density lipoprotein
PCR-RFLP: Polymerase chain reaction-fragment length polymorphism
Pro: Proline
ROS: Reactive oxygen species
rpm: Rotation per minute
SNP: Single nucleotide polymorphism
TC: Total cholesterol
TG: Triglycerides
UV: Ultraviolet
